# Artificial Intelligence-Driven Virtual Reality in Physical Rehabilitation: A Review of Machine-Learning Kinematics, Educational Scaffolding, and Treatment Adherence Strategies

**DOI:** 10.7759/cureus.110057

**Published:** 2026-06-01

**Authors:** Magalli Diaz Bravo

**Affiliations:** 1 Rehabilitation Science (AI and Digital Health), Independent Researcher, San Francisco, USA

**Keywords:** adherence to therapy, artificial intelligence (ai), digital therapeutics, educational technology, explainable artificial intelligence (xai), kinematic analysis, machine learning, physical medicine and rehabilitation (pm&r), telerehabilitation, virtual reality-based rehabilitation

## Abstract

Physical rehabilitation increasingly depends on interventions that are intensive, personalized, and sustained outside the clinic. Yet contemporary rehabilitation systems face persistent barriers, including workforce shortages, geographic inequities, rising costs, fragmented follow-up, and poor adherence to home exercise programs. This comprehensive review examines how artificial intelligence (AI) and virtual reality (VR) can function together as a digital therapeutic framework for physical rehabilitation.

The review argues that the core problem is not simply the absence of technology but the absence of continuous, meaningful supervision between clinic visits. AI-driven pose estimation, multimodal sensing, low-latency feedback, explainable analytics, and adaptive exercise progression allow rehabilitation programs to move from episodic observation to real-time, data-informed guidance. At the same time, principles of adult learning and motor learning help explain why immersion alone is not enough unless the system also teaches, motivates, and gradually transfers responsibility to the patient.

Across musculoskeletal, neurological, cardiovascular, oncological, and chronic pain populations, the evidence suggests that VR-supported rehabilitation can improve engagement, exercise capacity, movement quality, and patient satisfaction, particularly when paired with personalized coaching and home-based monitoring. This paper therefore proposes AI-VR rehabilitation not as a replacement for clinicians, but as a clinically governed co-pilot that extends supervision, strengthens adherence, and expands equitable access to therapy.

## Introduction and background

The delivery of physical rehabilitation in the United States is at a critical juncture. According to the most recent epidemiological data provided by the Centers for Disease Control and Prevention (CDC), disability affects approximately 28.7% of US adults, representing over 70 million individuals who reported having some type of functional disability in 2022. Within this population, mobility disability (serious difficulty walking or climbing stairs) affects 12.2% of the adult population [1-3]. Functional recovery after injury or chronic illness is not a passive event; it is a complex biological, behavioral, and educational process that requires repetition, feedback, and sustained participation over time [4,5]. However, the traditional clinic-based model of therapy is increasingly strained by practitioner shortages, transportation barriers, fragmented continuity of care, and escalating operational costs [6,7].

Current clinical research consistently identifies the central problem in rehabilitation as the adherence gap: patients may receive appropriate exercise prescriptions, but they often struggle to execute them consistently, accurately, and long enough to achieve meaningful functional change [7-9]. This gap is especially pronounced when patients leave the clinic and enter unsupervised environments where movement quality, motivation, symptom interpretation, and confidence fluctuate from day to day. In that context, the promise of artificial intelligence (AI) and virtual reality (VR) lies not merely in digitizing rehabilitation, but in restoring some of the supervision, interpretation, and encouragement that are normally lost between appointments [9-11].

Physical rehabilitation is also increasingly recognized as a public health and health systems priority rather than a narrow specialty service. Healthy People 2030 emphasizes access, participation, and support for people with disabilities across the healthcare system and the broader social environment [12]. The World Health Organization frames rehabilitation as an essential health service that should begin early, continue across the continuum of care, and help individuals maintain independence, work participation, and quality of life [4]. Consequently, AI-VR rehabilitation should be understood as part of a larger shift toward scalable, person-centered, technology-enabled care rather than as a stand-alone gadget or entertainment modality [4,11,13].

In this review, kinematics refers to the assessment of movement features such as joint position, trajectory, symmetry, and exercise form during rehabilitation tasks. Educational scaffolding refers to structured support that is provided more intensively at first and then gradually reduced as competence improves. In rehabilitation settings, machine-learning methods can help interpret movement data, detect deviations from prescribed exercise performance, and support more personalized feedback during home-based therapy. This review examines how these technical and educational components may work together to improve adherence and extend clinically meaningful supervision beyond in-person visits [9-11,13].

This comprehensive review delineates the technical, pedagogical, and clinical frameworks necessary for the successful implementation of AI-driven VR in physical rehabilitation. Specifically, this analysis examines: (1) the rehabilitation access crisis and the factors that make adherence decisive; (2) the technical architecture required for clinically useful AI-driven VR systems; (3) the educational foundations that make feedback therapeutically meaningful; (4) the application of these systems across multiple patient populations; and (5) the policy, safety, and research priorities necessary for responsible adoption.

## Review

Methodology and inclusion criteria

To ensure a broad synthesis of current work on AI-driven VR in rehabilitation, this article used an integrative narrative review approach. Literature was identified through PubMed/MEDLINE, Google Scholar, IEEE Xplore, and the Cochrane Library. The search focused on peer-reviewed articles, government reports, and technical standards published between 2019 and 2026, using terms such as “computer vision in physical therapy,” “human pose estimation,” “pedagogical scaffolding in clinical settings,” “telerehabilitation adherence,” and “interpretable machine learning.” Sources were considered for inclusion based on their relevance to the intersection of computer vision, motor learning theory, educational support, and clinical physical therapy. Duplicative or clearly non-relevant records were not retained. Ultimately, 31 sources, including clinical trials, systematic reviews, and official policy documents, were included in the narrative synthesis. Because this article is an integrative narrative review rather than a systematic review or meta-analysis, a formal PRISMA flow diagram, pooled quantitative synthesis, and formal risk-of-bias assessment were not performed.

The healthcare crisis in the United States and the expanding need for rehabilitation

Disability carries both functional and economic consequences. The current literature indicates that disabilities can sharply reduce household earnings and increase hidden costs such as transportation, home assistance, and dependency-related expenses [2,14]. These burdens do not affect health alone; they alter family economics, labor-force participation, and long-term social mobility. For many patients, incomplete rehabilitation therefore becomes both a clinical problem and a socioeconomic risk factor. When recovery stalls, individuals may require more prolonged care, depend more heavily on family caregivers, or struggle to return to school and employment [2,4,14].

The rehabilitation gap is intensified by workforce limitations. Data from the American Physical Therapy Association (APTA) reveal persistent shortages of rehabilitation professionals relative to the growing clinical need [6]. Even when services are available, patients frequently face waiting times, limited session numbers, transportation obstacles, and insurance constraints. Rural populations, low-income communities, and patients with severe mobility restrictions experience these barriers most acutely. In practical terms, many patients receive a front-loaded assessment and initial instruction, then are expected to maintain therapeutic intensity independently for weeks or months [4,6,13].

This mismatch between need and delivery is not unique to the United States. The WHO reports that approximately 2.4 billion people globally live with conditions that could benefit from rehabilitation and that unmet need remains substantial, particularly outside urban centers and in under-resourced settings. Rehabilitation is now recognized as a service that should be integrated into health systems at every level, not reserved for the end stage of treatment [4]. These observations strengthen the rationale for digital systems that can extend monitoring, teaching, and progression beyond hospital walls and outpatient facilities [13,15,16].

At the national level, technology-enabled rehabilitation aligns with public goals in disability inclusion, prevention of secondary complications, and health equity. When digital rehabilitation systems reduce missed visits, improve home exercise fidelity, and support continuity of care, they do more than enhance convenience. They address structural inequities in access and create a more resilient rehabilitation infrastructure capable of serving an aging population and a growing burden of chronic disease [3,4,12,16].

The adherence gap as the primary barrier to functional recovery

A critical insight within modern rehabilitation research is that even the most rigorous clinical plans fail to improve outcomes unless patients can consistently and accurately perform them. Home exercise adherence is historically poor, especially in musculoskeletal rehabilitation. Extensive connected health literature has demonstrated that nonadherence rates typically range from 30% to 50%, with certain cohorts exhibiting even lower rates of sustained compliance throughout the full episode of care [7,9]. Consequently, a significant proportion of therapeutic intent is lost once the patient transitions from the clinical setting to an unsupervised domestic environment.

Nonadherence is rarely a matter of willpower alone. It emerges from a convergence of factors: pain, fear of movement, uncertainty about correct technique, low self-efficacy, poor feedback, limited social support, weak goal ownership, and the monotony of repetitive exercise [5,9,11,17]. A patient may believe they are exercising consistently while performing insufficient repetitions, using compensatory patterns, or progressively reducing intensity because of doubt or discomfort. Traditional paper handouts and verbal reminders do not adequately address these barriers because they cannot observe performance, interpret deviation, or respond dynamically to patient behavior [7,9].

This is why adherence should be framed not only as a behavioral issue but as a design problem. If a rehabilitation system can provide collaborative goal setting, self-monitoring, real-time correction, educational coaching, graded difficulty, and visible progress, then adherence becomes more likely because the experience more closely resembles guided learning than isolated homework [9,17,18]. AI-VR platforms are uniquely positioned to operationalize these features simultaneously. In effect, they can transform home rehabilitation from an unsupervised assignment into an interactive, feedback-rich process that preserves therapeutic intent between clinician encounters [9-11,18].

For this reason, adherence is the hinge variable connecting technology, pedagogy, and public health. Improved adherence does not merely increase repetition counts. It creates the dosage, precision, and confidence required for neuroplastic adaptation, tissue loading, balance retraining, and cardiovascular conditioning. Therefore, the value proposition of AI-VR rehabilitation should be judged partly by whether it meaningfully changes treatment participation over time, not just whether it is novel or immersive [4,5,9,17].

Technical architecture and machine-learning-based kinematics

An effective AI-VR rehabilitation platform must combine precise movement capture with clinically interpretable feedback. Recent advancements in computer vision have established state-of-the-art single-camera systems capable of high keypoint accuracy and low inference latency, enabling real-time analysis of joint position and exercise form [1,19,20]. This technical foundation matters because therapeutic correction is time-sensitive. When cues are delivered too late, patients rehearse the very compensations clinicians seek to prevent; when feedback is immediate, the system can reinforce correct motor patterns while movement is still unfolding [19,20]. As outlined in Table 1, this sensing-analysis-learning workflow constitutes the functional backbone of the proposed AI-VR rehabilitation system, linking machine-learning kinematics, explainable AI (XAI), and educational scaffolding into a single closed-loop pipeline.

**Table 1:** Functional workflow of the AI-VR rehabilitation system. This cycle illustrates the transition from raw data capture to real-time pedagogical feedback. AI: artificial intelligence; VR: virtual reality.

Phase	Component	Action	Purpose
1. Sensing	Machine Learning Kinematics	Tracks 15+ biomechanical keypoints [1,20].	Objective data capture.
2. Analysis	Explainable AI (XAI)	Detects deviations from clinical norms [21].	Real-time clinical evaluation.
3. Learning	Educational Scaffolding	Provides "Knowledge of Performance" cues [18,22].	Enhances treatment adherence.

As shown in Table 1, the integration of these layers ensures that technical precision is translated into actionable educational guidance for the patient, moving beyond simple monitoring to a true learning intervention [4,18].

Pose estimation is especially attractive for home-based rehabilitation because it lowers the equipment burden. Markerless systems using RGB (red, green, and blue) cameras can estimate body landmarks without the cost and complexity of laboratory motion-capture infrastructure [1,20,23]. In rehabilitation settings, these models can quantify joint excursion, trunk alignment, gait asymmetry, squat depth, reach trajectories, and other movement features relevant to exercise quality. Recent AI reviews also emphasize that computer-vision systems can detect qualitative deviations that simpler repetition counters miss, including asymmetrical loading, compensatory trunk lean, poor spinal alignment, or inadequate terminal range [21,23].

The most clinically promising systems move beyond raw measurement toward a closed-loop architecture. In such a model, the patient performs within a VR or augmented digital environment; the camera and/or wearable sensors collect kinematic and physiological signals; the AI engine classifies performance quality and risk; and the platform delivers multimodal feedback through visual prompts, audio coaching, scores, or haptic cues [5,6,18]. This architecture supports not only error detection but also adaptive progression: successful performance can unlock more challenging tasks, whereas repeated compensations can trigger easier regressions, new demonstrations, or a clinician alert [20,21,23].

Importantly, accurate tracking alone is insufficient. Current clinical research emphasizes that XAI is a fundamental necessity for digital therapeutics, as medical professionals must understand the logic behind automated clinical recommendations [6]. Therapists need to know why the system flagged a movement and which biomechanical variables drove the recommendation. Without transparency, trust declines and integration into clinical decision-making becomes difficult. Explainable outputs, such as identifying knee valgus, reduced hip extension, asymmetrical weight shift, or excessive trunk substitution, allow the platform to mirror the reasoning structure of a therapist rather than functioning as an opaque score generator [21,23].

Multimodal sensing, data fusion, and longitudinal monitoring

Although camera-based pose estimation is central, the strongest systems will likely be multimodal. Wearable sensors, including inertial measurement units (IMUs), heart-rate devices, pressure sensors, and smart textiles, can complement computer vision by capturing movement velocity, load distribution, cadence, physiological stress, and exercise dose across time [13,15,23,24]. Multimodal fusion is especially useful when occlusion, lighting, or camera placement limits visual precision. It also broadens the scope of what can be monitored, allowing clinicians to pair movement quality with exertional response or symptom fluctuation [15,24].

Longitudinal monitoring is another major advantage. Traditional rehabilitation often depends on snapshots of performance during appointments. By contrast, AI-VR systems can generate repeated observations across days and weeks, allowing clinicians to track trends rather than isolated moments [13,20,23]. Recovery trajectories can then be modeled more intelligently: plateaus may be identified earlier, exercise progression can be personalized on objective data, and unexpected decline can prompt a timely reassessment before functional deterioration becomes entrenched. This temporal intelligence is one of the clearest ways digital therapeutics can extend the reach of expert care.

The intersection of pedagogy and rehabilitation

A foundational premise of this analysis is that physical rehabilitation must be viewed fundamentally as an educational process. Patients are not simply completing physical tasks; they are engaged in a complex pedagogical journey of relearning movement, recalibrating effort, interpreting bodily signals, and building confidence in their own physical capacity [4,5,18,22]. From this perspective, AI precision is clinically useful only when the feedback is designed in ways that patients can understand, apply, and internalize. A technically sophisticated platform that overwhelms, confuses, or discourages the user will not improve outcomes, no matter how accurate its kinematic model may be.

Adult learning theory is therefore highly relevant. Andragogy emphasizes autonomy, relevance, self-direction, and immediate applicability. In rehabilitation, adult learners are more likely to engage when the purpose of a task is clear, the goal aligns with daily life, and progress is visible [7,22]. VR environments can operationalize these principles by embedding exercises in meaningful scenarios, such as reaching for household objects, stepping over obstacles, navigating community-like spaces, or completing work-simulated tasks, rather than presenting movement as abstract repetition [10,11,18].

Motor learning theory further strengthens this argument. Effective recovery depends on repetition, feedback timing, task specificity, and graded challenge. Knowledge of performance (KP) helps patients understand how they moved; knowledge of results (KR) helps them understand what outcome they achieved [5,18]. AI-VR systems can integrate both. For example, a patient may receive KP in the form of an avatar-based cue about trunk alignment and KR through a score reflecting successful completion of a reaching task. This dual channel can make rehabilitation more cognitively coherent and behaviorally reinforcing than conventional handout-based home exercise [5,17,18].

The educational value of VR also extends to self-management. Patients recovering from chronic or recurrent conditions must often learn pacing, safe progression, symptom interpretation, and relapse prevention. Interactive digital environments can deliver this information contextually rather than as detached advice. In this sense, AI-VR platforms can function not only as exercise monitors but also as teaching systems that translate clinical knowledge into structured patient action [9,10,18,22].

Educational scaffolding, self-efficacy, and behavioral persistence

Scaffolding is the process of providing support intensively at first and then gradually withdrawing it as competence improves. In digital rehabilitation, this concept is especially powerful. Early in recovery, patients may need frequent demonstrations, simplified tasks, explicit visual cues, slower pacing, and reassurance about errors. As performance stabilizes, the system can reduce cue density, increase complexity, and promote self-correction. This adaptive tapering is more educationally sophisticated than static feedback because it respects the learner’s developmental stage rather than treating all users identically [18,22].

Self-efficacy is equally central to therapeutic success. Current clinical research identifies an individual's confidence in their ability to perform exercises as a major predictor of long-term adherence [17]. Patients who experience small, visible wins are more likely to continue, whereas repeated uncertainty or perceived failure can trigger disengagement. AI-VR systems can support self-efficacy by setting achievable challenge levels, visualizing progress, reinforcing successful performance, and linking exercises to meaningful functional goals. These mechanisms help patients reinterpret rehabilitation from a sequence of burdensome tasks into a progressive skill-building process [9,17,18].

Graded exposure is another important pedagogical and psychological strategy. Many patients, particularly those with chronic pain, post-surgical apprehension, or neurological instability, develop fear of movement (kinesiophobia). VR environments allow clinicians to simulate tasks in ways that feel safe, controllable, and repeatable. A patient may begin with simplified virtual tasks, progress to more demanding scenarios, and gradually rebuild tolerance and trust in movement. This is therapeutically significant because fear often suppresses movement quality, exercise dosage, and confidence even when the tissue or neurological state would permit greater participation [5,11].

Gamification contributes to this process when used thoughtfully. Scores, levels, missions, and rewards can sustain attention and make repetition more tolerable, but they must remain clinically aligned. The goal is not entertainment for its own sake; it is sustained therapeutic engagement [9,10,17]. When game mechanics are tethered to functional goals and adaptive difficulty, they can increase duration, repetition volume, and intrinsic motivation. When poorly designed, however, they may distract from movement quality or create superficial engagement without transfer. Thus, gamification should be considered a pedagogical instrument rather than a decorative layer [17].

Clinical applications and specialized care pathways

The clinical value of AI-VR systems extends beyond any single diagnostic group. The primary strength of these platforms lies in their inherent adaptability: a unified architecture can support diverse exercise libraries, progression rules, safety thresholds, and outcome measures tailored to the specific needs of different patient populations [5,10,11]. This versatility positions AI-VR rehabilitation not only as a localized treatment technology but also as a highly scalable service model. The following sections examine several major application domains in greater detail.

Musculoskeletal rehabilitation

Musculoskeletal rehabilitation is one of the most immediate use cases because it frequently depends on home exercise and movement-quality correction between appointments. Patients with musculoskeletal conditions are often prescribed home exercise programs that must be performed independently for meaningful recovery to occur [8,9,13]. AI-VR systems can monitor exercise fidelity, count repetitions, measure range, and identify compensation patterns that would otherwise go unnoticed until the next visit [20,21,23].

Digital musculoskeletal care frameworks increasingly emphasize that remote rehabilitation should include structured preparation, safety screening, standardized self-report measures, and clear triage pathways for when in-person care is needed. This perspective fits naturally with AI-VR models. A clinician can prescribe an initial program, the platform can supervise day-to-day execution, and deviations from expected progress can trigger asynchronous review or hybrid follow-up. In this model, technology does not eliminate professional judgment; it multiplies its reach [13].

Neurological and stroke rehabilitation

Neurological rehabilitation, especially post-stroke care, is another major domain of interest because recovery depends heavily on repetition, task specificity, and neuroplastic adaptation. VR environments are well suited to repetitive task practice because they can simulate activities of daily living, increase engagement, and provide consistent feedback [5]. Recent systematic evidence on home-based VR training for upper extremity recovery after stroke indicates positive effects on motor outcomes, particularly when systems are customized for clinical use and deliver real-time feedback with adaptive difficulty [25]. Beyond upper-extremity recovery, lower-extremity strength and endurance are equally decisive for functional independence after stroke, and progressive resistance training principles previously validated in cardiovascular populations have demonstrated transferable benefits on muscular performance and exercise tolerance that can inform neurorehabilitation protocols [25,26].

The implementation implications are important. AI-VR platforms should therefore be built to map digital performance metrics onto recognized clinical endpoints rather than inventing isolated technical scores. This alignment increases interpretability, supports clinician adoption, and strengthens research comparability across trials [5,25].

Cardiovascular rehabilitation

Cardiovascular rehabilitation illustrates the dual capacity of VR to influence both physical performance and emotional engagement. Current clinical evidence specifically emphasizes the efficacy of tailored exercise prescriptions for populations managing heart disease and heart failure [26,27]. More recent evidence from a systematic review and meta-analysis shows that VR-based cardiac rehabilitation can improve exercise capacity and reduce anxiety, depression, and stress-related outcomes [28]. These findings are clinically meaningful because adherence to cardiac rehabilitation is often compromised by fear, low motivation, transportation barriers, and the emotional consequences of major cardiac events.

In an AI-VR framework, cardiovascular applications could combine movement analysis with physiological monitoring, exertion thresholds, symptom prompts, and safety alerts. Rather than delivering generic exercise videos, the system could titrate intensity, watch for form deterioration under fatigue, and provide reassurance or escalation pathways when symptoms emerge. Such a model would be especially useful for extending supervised exercise into home settings while maintaining clinician oversight [24,27,28].

Oncology rehabilitation

Exercise is established as a clinically vital intervention in oncology populations, particularly for managing symptoms such as cancer-related fatigue, deconditioning, and ICU-acquired weakness [26,29]. Emerging evidence now suggests that VR-based exercise rehabilitation is a feasible and promising option for patients with cancer-related dysfunctions, with reported benefits in limb function, range of motion, edema, activities of daily living, and quality of life [30]. High compliance and satisfaction rates are particularly relevant because cancer rehabilitation often competes with symptom burden, treatment fatigue, and the psychological stress of ongoing care [29,30].

For oncology populations, educational design is essential. Patients may need clear instructions about pacing, safety, symptom monitoring, and the rationale for movement even during intensive treatment periods. AI-VR systems can help personalize this process by modifying task intensity to daily symptom status, embedding recovery education into the exercise environment, and highlighting meaningful achievements rather than maximal performance. In this way, technology supports both functional maintenance and emotional persistence [29,30].

Chronic pain, fear of movement, and behavioral rehabilitation

Chronic pain rehabilitation benefits from interventions that address both physical capacity and fear-based avoidance. In these populations, pain experience, movement apprehension, catastrophizing, and low confidence can reduce participation even when a graded exercise approach is indicated [5,11,13]. VR offers an unusual therapeutic advantage because it can recontextualize movement, reduce threat, and direct attention toward goal achievement rather than symptom anticipation.

When paired with AI-based form monitoring, VR can support graded exposure with safety-sensitive progression. Patients can practice movement in structured scenarios that reinforce success, while clinicians receive data on whether the patient is tolerating, avoiding, or compensating through the task. This could be especially valuable in populations where symptom reporting alone does not fully capture functional behavior. As a result, chronic pain care may become less reactive and more behaviorally informed [11,13,23].

Comparative analysis of modalities

To evaluate the practical efficacy of technology-enabled care, it is necessary to compare the proposed AI-VR framework against existing delivery models. Traditional clinic-based therapy and unsupervised home exercise represent the current poles of rehabilitation, yet both face significant limitations in scalability and consistent feedback. Table 2 provides a literature-informed conceptual comparison of three rehabilitation delivery modalities, i.e., traditional clinic-based therapy, unsupervised home exercise, and the proposed AI-VR system, across clinical, technical, and economic domains. The listed values summarize patterns reported in the literature rather than pooled statistical estimates and illustrate how an integrated digital therapeutic platform may combine the accessibility of home care with the high-fidelity supervision typically associated with in-person clinical visits [4,6,7,9].

**Table 2:** Comparative analysis of rehabilitation delivery modalities across clinical, technical, and economic metrics. AI: artificial intelligence; VR: virtual reality.

Criterion	Clinic-based therapy	Unsupervised home exercise	Proposed AI-VR system
Accessibility	Low (Travel & Cost)	High (Domestic)	High (Domestic)
Feedback Frequency	Occasional (Weekly)	None (Static)	Continuous (Real-time) [19,20]
Adherence Rate	High (Supervised)	Extremely Low (<30%)	Optimized (>85%) [7,9]
Data Fidelity	Subjective [14]	None (Self-reported)	Objective (Kinematic) [1,31]
Safety/Supervision	High (Human)	Low (Unsafe)	High (Digital Supervisor) [16,31]
Cost-Effectiveness	Low (High Labor)	High (Low Cost)	Optimized (Scalable) [4,6]
Educational Input	High (In-person)	None	High (Scaffolding) [18,22]

This comparison highlights the "adherence gap" inherent in unsupervised models and demonstrates how the proposed AI-VR system utilizes real-time kinematics and educational scaffolding to mirror clinical-grade supervision in a domestic setting.

Implementation, workflow integration, and system design

A major determinant of success will be whether AI-VR rehabilitation platforms fit real clinical workflows. Digital care should not be conceived as a parallel universe disconnected from therapists’ reasoning processes. Instead, systems should support screening, baseline assessment, exercise prescription, remote monitoring, asynchronous review, and timely escalation when recovery deviates from expectation. In this regard, AI is best understood as a clinical co-pilot: it extends observation and surfaces relevant patterns, but the clinician retains interpretive authority and responsibility for treatment planning [13,23].

Practical implementation begins with a robust setup. Patients need adequate space, lighting, safe flooring, stable internet when required, and accessible interfaces. Clinicians need dashboards that synthesize movement data, patient-reported symptoms, adherence logs, and outcome trajectories into a format that is quick to interpret [9,13,15]. Without these workflow supports, even accurate technology can become burdensome rather than helpful.

Communication design is equally important. Remote environments lack tactile correction and many of the implicit cues of in-person care, so instructions must be visually clear, verbally precise, and contextually timed [13]. Automated prompts should feel clinically purposeful rather than generic. For some patients, asynchronous check-ins, short educational modules, and performance summaries may be more effective than frequent live sessions. For others, especially those at high risk, post-operative, or neurologically complex, a hybrid model combining digital supervision with periodic in-person evaluation may be safest and most effective [11,13].

Interoperability and reimbursement will also shape adoption. If digital rehabilitation platforms remain separate from electronic documentation, billing pathways, and established clinical outcome measures, implementation will stall even when efficacy is promising. Future-ready systems should therefore prioritize standards-based data integration, transparent documentation, and features that help clinicians justify progression, intensity, and clinical decisions using objective evidence [13,24].

Ethics, safety, accessibility, and trust

The expansion of AI into home rehabilitation introduces important ethical and practical concerns. Vision-based systems collect sensitive biomechanical and sometimes identifiable visual data, while wearable sensors may generate continuous streams of behavioral and physiological information. This raises questions about privacy, consent, storage, algorithmic bias, and secondary data use [21,23]. Systems used in healthcare must therefore be designed with explicit security protections and communication that patients can understand.

Bias and generalizability are equally important. AI models trained on narrow datasets may perform differently across body types, skin tones, ages, clothing styles, assistive-device use, and household environments [27]. If not addressed, these limitations could undermine both accuracy and equity. The same populations most likely to benefit from remote rehabilitation, including older adults, rural patients, lower-income households, and people with complex disability, may also be those most vulnerable to digital exclusion if interfaces are overly technical or if broadband access is assumed [3,12,13,16].

Safety protocols should also be embedded directly into platform design. Remote rehabilitation demands fall-risk awareness, symptom check prompts, emergency escalation plans, and clearly bounded task progression [13,27]. For cardiovascular, post-operative, and oncology populations especially, the system should incorporate clinician-defined red flags and intensity thresholds rather than relying on generic progression. Ethical deployment therefore requires more than data protection; it requires clinically bounded autonomy.

Outcome measurement and research design priorities

To mature as a field, AI-VR rehabilitation must be evaluated with outcome frameworks that are both clinically meaningful and technologically specific. Functional improvement should remain central and may include mobility, strength, endurance, range of motion, gait quality, upper-extremity dexterity, activities of daily living, symptom burden, and return-to-work or participation outcomes [4,5,25-30]. At the same time, digital systems generate additional metrics, such as movement quality scores, response latency, session duration, adherence trajectories, and error patterns, that can enrich interpretation when aligned with established clinical scales.

The literature also needs better trial design. Several of the recent reviews identified heterogeneity in intervention types, small sample sizes, limited long-term follow-up, and inconsistent outcome reporting [23,25,28,30]. Future studies should compare AI-VR rehabilitation not only with no-treatment controls but also with standard home exercise, conventional telerehabilitation, and hybrid clinician-guided models. It will be especially important to determine which patient subgroups benefit most, how much therapist oversight is required, and whether improved short-term engagement translates into durable functional gain.

Economic evaluation should be included as well. Given workforce shortages and disability-related costs, policymakers and health systems will want to know whether AI-VR systems reduce readmissions, shorten recovery timelines, improve productivity, or offset therapist scarcity through more efficient monitoring [2,4,6,14]. Research that combines clinical outcomes with implementation and cost-effectiveness data will be especially persuasive for large-scale adoption.

Discussion: National importance and public health impact

The national importance of AI-driven VR rehabilitation lies in its ability to connect several problems that are usually treated separately: disability burden, health-workforce limitations, weak home-exercise adherence, digital inequity, and rising chronic disease prevalence [2,4,6,12]. By improving supervision and engagement outside the clinic, AI-VR systems may help reduce preventable functional decline and secondary complications while preserving clinician time for patients who most need direct intervention.

The public-health significance is especially strong when these systems are viewed through an equity lens. A technology that enables objective monitoring at home can reduce the penalty currently imposed by geography, transportation barriers, and limited local specialist supply [4,13,16]. If designed accessibly and governed responsibly, AI-VR rehabilitation can support people who have historically faced fragmented or inconsistent care. This aligns with national goals in disability inclusion, healthy aging, and digital health innovation [3,12,16].

At the same time, the field should avoid technological determinism. AI-VR rehabilitation will not succeed merely because the components are novel. Its value depends on pedagogy, safety, clinician governance, workflow integration, and equitable implementation. The strongest future systems will likely be those that combine machine intelligence, immersive design, and human clinical judgment into a coherent therapeutic ecosystem rather than privileging any one component in isolation [11,13,18,21,23].

## Conclusions

The convergence of AI, VR, and educational science represents a robust and timely response to the modern rehabilitation crisis. The fundamental challenge confronting the healthcare system is not merely the prescription of exercise, but the maintenance of accurate, motivating, and clinically meaningful practice once a patient transitions to an unsupervised domestic environment. AI-supported VR systems may help address this adherence gap by combining motion analysis with adaptive feedback and structured therapeutic support. By transforming rehabilitation into an immersive and supportive educational journey, these technologies ensure that therapeutic intent is preserved throughout the full episode of care, regardless of the patient's physical distance from a clinical facility.

When implemented within a structured clinical framework, these systems possess the capacity to extend specialist expertise and personalize recovery trajectories across diverse populations, including those managing musculoskeletal, neurological, cardiovascular, oncological, and chronic pain conditions. They are best understood not as replacements for the multidisciplinary rehabilitation team, but as digital therapeutic partners that bridge the divide between clinical appointments and daily life. Future progress in this field will depend on the establishment of transparent analytics, interoperable workflows, and policy frameworks that prioritize equitable access to innovation. Ultimately, integrating immersive rehabilitation technologies with motor learning principles may support more effective and scalable models of physical rehabilitation.
